# RNA N6-methyladenosine demethylase FTO promotes osteoporosis through demethylating Runx2 mRNA and inhibiting osteogenic differentiation

**DOI:** 10.18632/aging.203377

**Published:** 2021-09-08

**Authors:** Jing Wang, Qiang Fu, Jian Yang, Jin-Long Liu, Shu-Ming Hou, Xing Huang, Jia-Shi Cao, Tie-Long Liu, Kun-Zheng Wang

**Affiliations:** 1 Department of Orthopedics, The Second Affiliated Hospital of Xi’an Jiaotong University, Xi’an, Shaanxi, China; 2 Spinal Tumor Center, Chang Zheng Hospital, Second Military Medical University, Shanghai, China; 3 Department of Orthopedic Trauma Surgery, Second Military Medical University, Shanghai, China; 4 Department of Biotechnology and Pathology, School of Medical Technology, Shanghai University of Medicine and Health Sciences, Shanghai, China

**Keywords:** osteoporosis, N6-methyladenosine (m6A), FTO, bone marrow mesenchymal stem cells

## Abstract

As a systemic disease, osteoporosis (OP) results in bone density loss and fracture risk, particularly in the hip and vertebrae. However, the underlying molecular mechanisms of OP development have not been fully illustrated. N6-Methyladenosine (m6A) is the most abundant modification of mRNAs, which is involved in many of pathological processes in aging disease. However, its role and regulatory mechanism in OP remains unknown. Here, we aimed to investigate the roles of m6A and its demethylase FTO in OP development. The results showed that m6A methylated RNA level was up-regulated in the bone marrow mesenchymal stem cells (BMSCs) from patients with OP. The level of N6-methyladenosine demethylase FTO was consistently decreased in the BMSCs from patients with OP. Functionally, lentivirus-mediated FTO overexpression in normal BMSCs to compromised osteogenic potential. Mechanism analysis further suggested that FTO overexpression decreased the m6A methylated and total level of runt related transcription factor 2 (Runx2) mRNA, subsequently inhibited osteogenic differentiation. We found that FTO inhibition could effectively improve the bone formation in ovariectomized osteoporotic mice *in vivo*. Together, these results reveal that RNA N6-methyladenosine demethylase FTO promotes osteoporosis through demethylating runx2 mRNA and inhibiting osteogenic differentiation.

## INTRODUCTION

Osteoporosis (OP) is a common bone disease regardless of gender [1], which is characterized by low bone mass and damaged bone structure, which can lead to increased risk of fracture [2]. Previous evidence indicated that OP-induced factures are usually associated with higher mortality and can limit patient activity, thus increasing medical burden in China [3, 4]. As multipotent progenitor cells, BMSCs can be differentiated into adipogenic, chondrogenic and osteoblastic lineages [5]. It is well documented that the abnormal lineage differentiation of endogenous BMSCs leads to osteoporosis [6]. However, the underlying molecular mechanisms by which BMSCs in OP failed with osteogenic differentiation needs to be further studied, which hinders the clinical treatment of osteoporosis.

N6-Methyladenosine (m6A) modification, which is catalyzed by a methyltransferase complex including of METTL3, METTL14 and their cofactors [7], WTAP, VIRMA, and RBM15 [8], is one of the most common modifications of mRNAs in eukaryotes [9, 10]. It is usually reversed by two mammalian RNA demethylases, ALKBH5 and FTO [11, 12]. These methyltransferases and demethylases maintain the dynamic hemostasis of m^6^A methylation in mammalian cells. FTO is involved in a variety of pathological processes by demethylating its target mRNAs. Recent *in vitro* and *in vivo* studies have led to major breakthroughs in deciphering the interesting role of m^6^A in mammalian development [13]. Abnormal levels of m^6^A during FTO elevation attenuates cell cycle progression, disrupts normal lineage commitment and functional stem cell differentiation, leading to neurogenesis retardation, immune deficiency, and sterility [10, 14, 15]. However, the actual roles and underlying molecular basis of FTO in the osteogenic differentiation disorders of BMSCs and the OP development remains unknown.

Considering the strong correlation between FTO and health or disease, we focused on the potential role of FTO in impaired osteogenic differentiation of OP, but little is known about it. Here, we demonstrated that the m^6^A methylated RNA level were up-regulated in the BMSCs from patients with OP (OP-BMSCs). The level of N6-methyladenosine demethylase FTO was consistently decreased in the OP-BMSCs group. Functionally, FTO overexpression in normal hBMSCs compromised osteogenic potential. Mechanism analysis further demonstrated that FTO overexpression decreased the m^6^A methylated and total level of runt related transcription factor 2 (RUNX2) mRNA, subsequently inhibited osteogenic differentiation. Moreover, we found that FTO inhibition could effectively improve the bone formation in ovariectomized osteoporotic mice *in vivo*. Taken together, these results reveal that RNA N6-methyladenosine demethylase FTO promotes osteoporosis through demethylating RUNX2 mRNA and inhibiting osteogenic differentiation.

## MATERIALS AND METHODS

### Patient and clinical samples with ethical approval

A total of 40 cases of BMSCs with written informed consent were collected (20 cases from bone fracture patients, 20 OP cases) from patients admitted to Changzhen Hospital (Shanghai, China) between July 2016 and July 2019. Moreover, this study was approved by the Ethics Committee of Changzhen Hospital (Shanghai, China), and the consent of the patients was obtained before enrollment.

### m6A RNA methylation quantification

We used the m^6^A RNA Methylation Assay Kit (Abcam, ab185912) to detect the total of m^6^A in total RNA as previously reported [12].

### Quantitative real-time PCR (qRT-PCR)

A total of 1 μg of RNA is used in cDNA synthesis using TRIzol reagent. Real-time PCR was performed using ABI Vii7 system (Applied Biosystems, USA). Relative gene expression is by comparison of the comparative CT method (ΔΔCT).

### Ovariectomized (OVX) animal model

The ovariectomized (OVX) animal model was generated as previous report [16]. Briefly, female C57BL/6J mice (14 weeks old) were treated with bilateral ovariectomy under general anesthesia. Eight weeks after surgery, tibial plateau was harvested and the structure were evaluated with a SCANCO Medical μCT 40 scanner. The three-dimensional structural parameters was analyzed as following: TV (total tissue volume, containing both trabecular and cortical bone), bone volume/tissue volume ratio (BV/TV), the trabecular number (Tb.N) and trabecular thickness (Tb.Th), while decreasing the trabecular separation (Tb.Sp) and structure model index (SMI). After the m-CT scan, the femurs were dehydrated and embedded in methyl methacrylate. Five μm thick slices were prepared using Leica RM2235 slice mechanism and stained by H&E.

### Western blot

Antibodies against FTO, RUNX2 and GAPDH (Abcam, Cambridge, MA, USA) were used in the Western blotting as previously described [17].

### Cell culture

BMSCs were cultured in α-MEM medium (Thermo Fisher, Gibco) containing including 10% FBS (Thermo Fisher, Gibco) and 1% penicillin and streptomycin (Thermo Fisher, Gibco) at 37 ° C in the presence of 5% CO_2_.

### Lentiviral transduction overexpression studies

FTO gene was constructed into pLVX-IRES-ZsGreen plasmid. The pLVX-IRES- ZsGreen-FTO construct were transfected into the HEK293T cells together with the psPAX2 and pMD2.G plasmids. The viral supernatant was collected after 48 hours of transfection and used for the infection of MSCs.

### Cell proliferation assay

The cell proliferation was measured by Cell Counting Kit-8 (CCK-8, Beyotime, Shanghai, China) as previously described [12].

### Alizarin red staining, ALP staining and ALP activity assay

Alizarin red staining (ARS), ALP staining and ALP activity assay were performed as report previously [16].

### Me-RIP assay

The methylated Runx2 RNA was purified by the methylated m6A RNA immunoprecipitation (me-RIP) and evaluated by qRT-PCR as described previously [12].

### Statistical analysis

The data were expressed as mean ±SEM. The comparison between the two groups was performed by unpaired, double-tailed T-test. For multiple comparisons, ANOVA or repeat ANOVA was used, and then Bonferroni post mortem was performed using GraphPad Prism® 9.0 software. P < 0.05 was considered statistically significant.

## RESULTS

### The level of methyltransferase FTO is elevated in BMSCs from OP patients and OVX mouse

**Figure 1:**
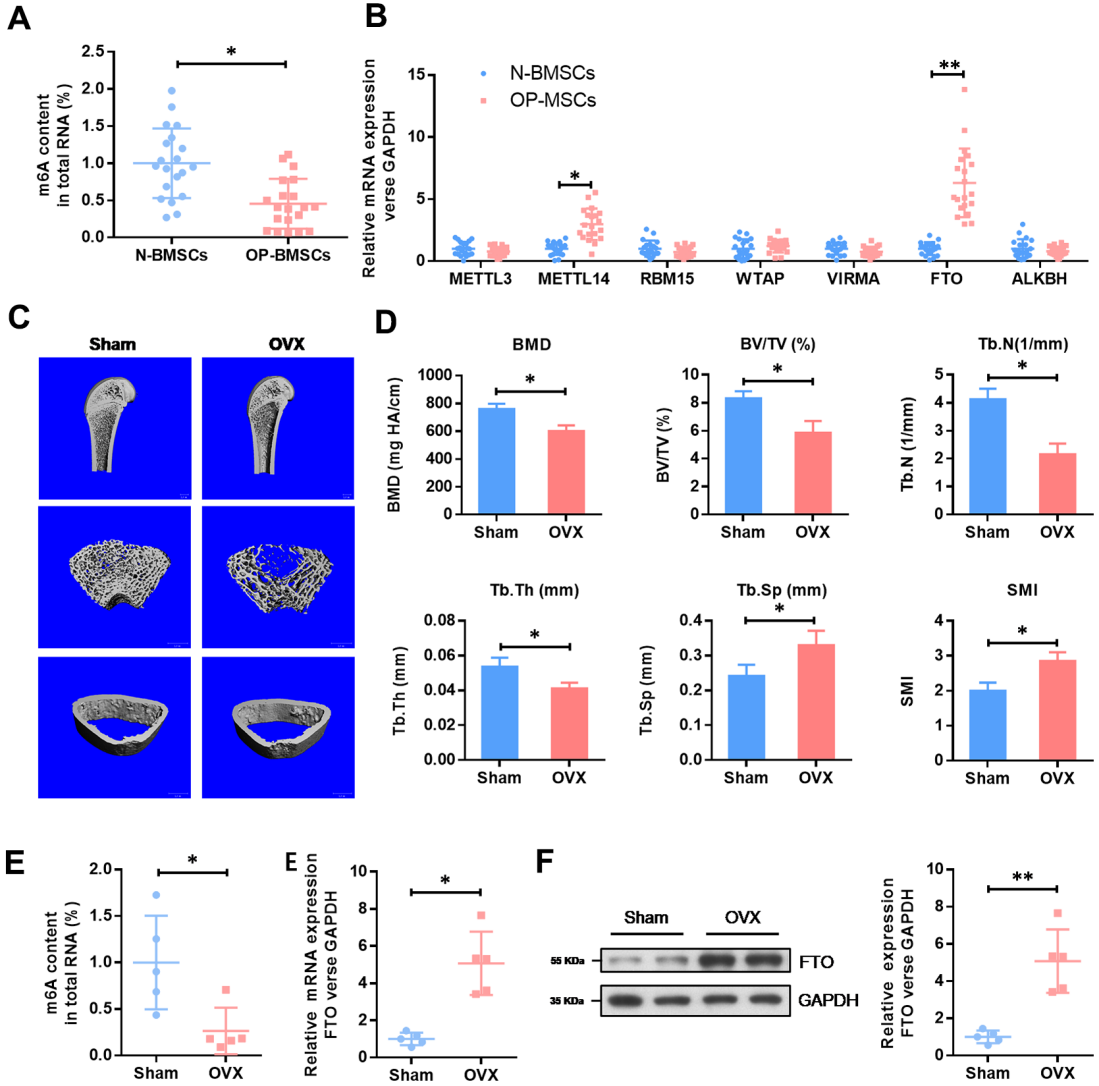
**The expression level of methyltransferase FTO is elevated in BMSCs from OP patients and OVX mouse.** (**A**) The methylated RNA (m6A) level in 20 cases of normal BMSCs from bone fracture patients (N-BMSCs) and 20 cases of BMSCs from OP patients (OP-BMSCs). *P<0.05. (**B**) The mRNA level of critical methyltransferase (METTL3, METTL14, RBM15, WTAP and VIRMA) and demethylases (FTO and ALKBH1) in 20 cases of normal BMSCs from bone fracture patients (N-BMSCs) and 20 cases of BMSCs from OP patients (OP-BMSCs) were analyzed by qRT-PCR. *P<0.05 and **P<0.01. (**C**) Representative images of μCT reconstructive images of tibial plateau in sham and OVX groups. (**D**) 3D structural parameters-BMD, BV/TV, Tb.N, Tb.Sp, Tb.Th and SMI-of tibial plateau by μCT in sham and OVX groups. (**E**) The methylated RNA (m6A) level and FTO expression in BMSCs from sham and OVX mouse. *P<0.05. (**F**) The protein level of FTO in BMSCs from sham and OVX mouse were analyzed by western blot. **P<0.01.

To understand the role of m^6^A in OP, we evaluated the total methylated m^6^A RNA levels in human BMSCs. We collected 20 cases of normal BMSCs from bone fracture patients (N-BMSCs) and 20 cases of BMSCs from OP patients (OP-BMSCs). As shown in the Figure 1A, the total m^6^A methylated RNA level was remarkedly down-regulated in OP-BMSCs compared with N-BMSCs. Next, we evaluated the mRNA levels of major methyltransferase (METTL3, METTL14, RBM15, WTAP and VIRMA) and demethylases (FTO and ALKBH1). These results showed that the mRNA level of FTO was significantly elevated in OP-BMSCs (Figure 1B).

Next, we generated an osteoporotic mouse model with OVX-induced OP. Consistent with an increase in SMI and Tb.Sp, a significant decrease in the parameters of BMD, BV/TV, Tb.N and Tb.Th in OVX groups were found by μCT. These results demonstrated an osteoporotic phenotype in OVX mice. In accordance with the results in human BMSCs, the total m^6^A methylated RNA level was remarkedly down-regulated in OVX-BMSCs, compared with Sham-BMSCs, while the expression level of FTO was elevated obviously (Figure 1E). The increased expression of FTO protein in OVX-BMSCs was further confirmed by western blot (Figure 1F). These results evidenced that FTO may be involved in the development of OP via m6A modification.

### FTO over-expression impairs the BMSCs osteogenic differentiation

**Figure 2:**
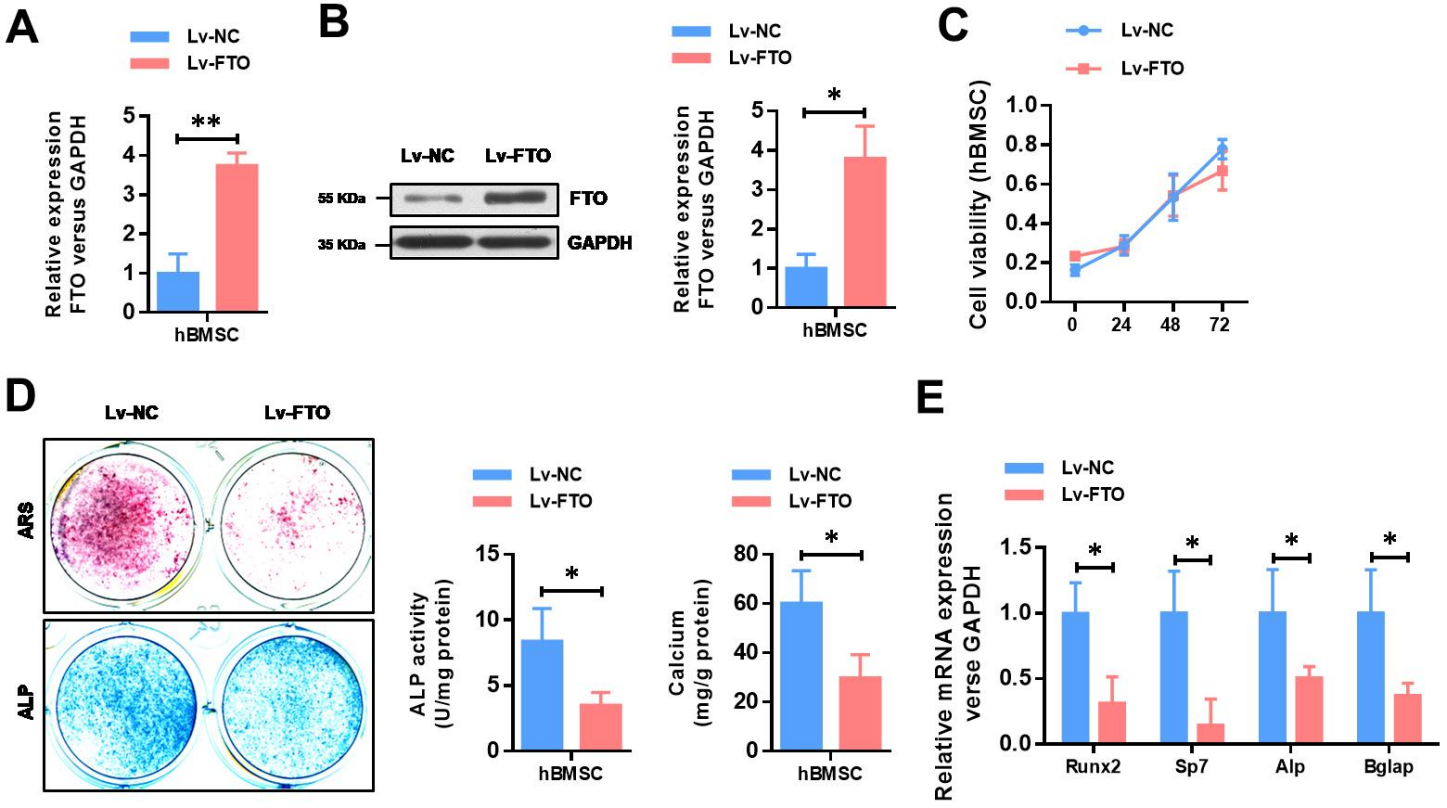
**FTO over-expression impairs the osteogenic differentiation of BMSCs.** (**A**) The efficiency of FTO over-expression in BMSCs were confirmed by quantitative real-time PCR. *P<0.05. (**B**) The efficiency of FTO over-expression in BMSCs were confirmed by western blot. *P<0.05. (**C**) The cell viability of BMSCs were analyzed by CCK8 assay. (**D**) Representative images and quantitative analyses of ALP and ARS staining of MSCs after 14 days of osteogenic induction. (**E**) qRT-PCR analyses of the expression of Runx2, Sp7, Alp and Bglap in MSCs after 7 days of osteogenic induction. *P<0.05.

To further explore the functional role of FTO in OP, we over-expressed the expression of FTO in hBMSCs. The efficiency of FTO overexpression in hBMSCs cells were confirmed by qRT-PCR (Figure 2A) as well as western blot (Figure 2B). Then, cell proliferation assay indicated that FTO over-expression did not affect the proliferation ability of hBMSCs (Figure 2C). To investigate its role in in osteogenesis of MSCs, osteogenic differentiation was conducted. Reduced activity of alkaline phosphatase staining (ALP), decreased calcium mineralization (Figure 2D), and down-regulated expression of osteogenic markers, including Runx2, Sp7, ALP, and Bglap (Figure 2E) indicated impaired osteogenic differentiation in FTO-overexpressed mesenchymal stem cells. These results indicated that FTO overexpression damaged osteogenic differentiation of BMSCs.

### FTO represses the expression of Runx2 through its m6A demethylase activity

**Figure 3:**
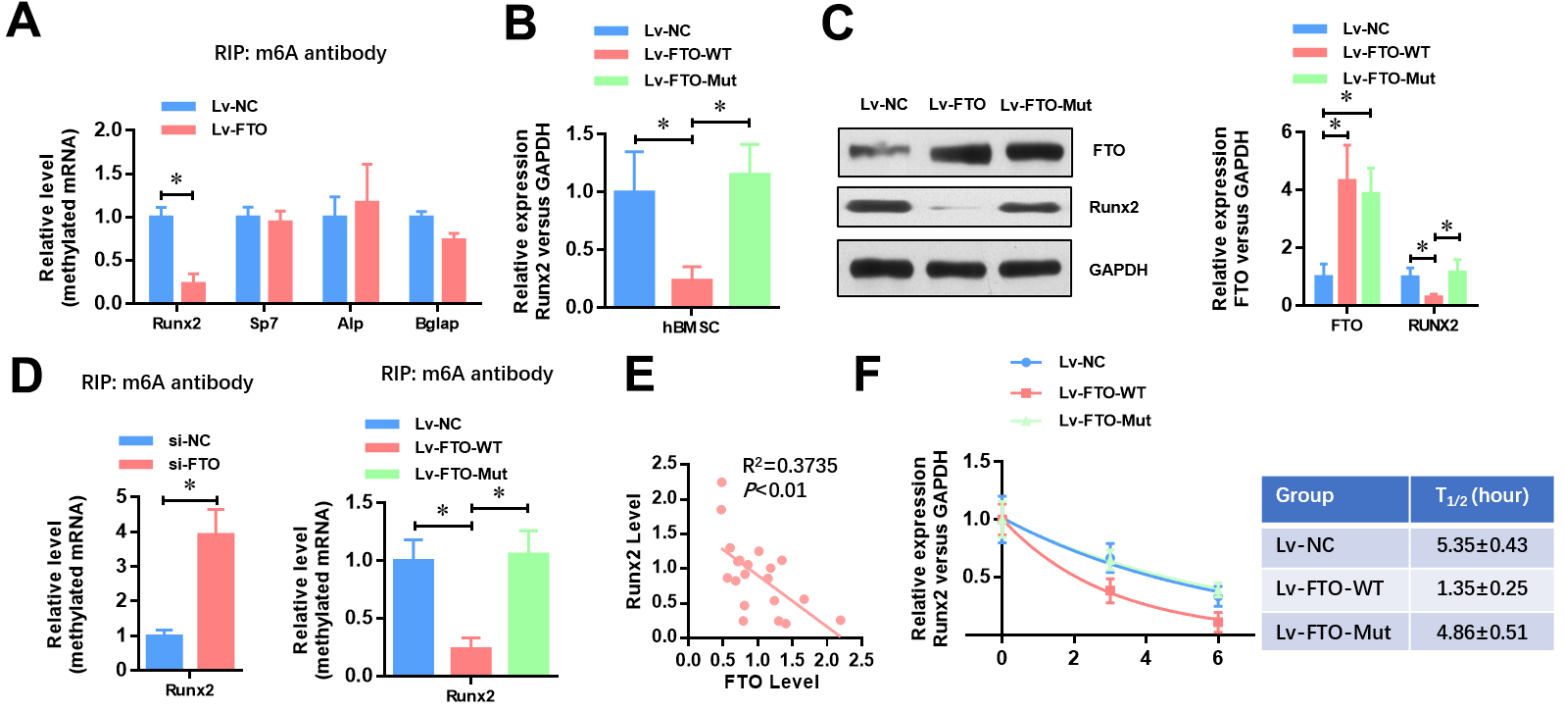
**FTO represses the expression of Runx2 through its m6A demethylase activity.** (**A**) The methylated RNA (m6A) level of Runx2, Sp7, Alp and Bglap in normal and FTO over-expressed BMSCs. *P<0.05. (**B**) The mRNA level of Runx2 in Wild-type or demethylase mutated FTO (R96Q) over-expressed BMSCs. *P<0.05. (**C**) The protein level of Runx2 in wild-type or demethylase mutated FTO (R96Q) over-expressed BMSCs. *P<0.05. (**D**) The methylated RNA (m6A) level of Runx2 in FTO down-regulated, wild-type or demethylase mutated FTO (R96Q) over-expressed BMSCs. *P<0.05. (**E**) Correlation between the mRNA level of FTO and Runx2 in the 20 cases of OP patients (R^2^=0.3735, *P*<0.01, Pearson’s correlation test). (**F**) Half-life of Runx2 mRNA in FTO down-regulated, wild-type or demethylase mutated FTO (R96Q) over-expressed BMSCs after transcription inhibition (TI). All values were normalized to 18S rRNA.

To identify the underlying mechanisms of FTO impaired osteogenic differentiation, we analyzed whether the methylated m6A level of osteogenic markers (Runx2, Sp7, Alp and Bglap) was affected by FTO. As shown in Figure 3A, FTO over-expression dramatically down-regulated the methylated level of Runx2 mRNA (Figure 3A). Moreover, FTO over-expression significantly down-regulated the mRNA (Figure 3B) and protein (Figure 3C) levels of Runx2 in hBMSCs. Next, we generated a mutated FTO (R96Q) construct with disordered enzymatic activity as described previously [12]. The result showed that the mRNA and protein levels of Runx2 was not affect when the mutated FTO (R96Q) was overexpressed in hBMSC. Meanwhile, the methylated level of Runx2 mRNA was increased by FTO down-regulation whereas wildtype FTO but not mutated FTO overexpression decreased the methylated mRNA level of Runx2. Moreover, the mRNA level of FTO and Runx2 showed a negative correlation in the 20 cases of OP patients (Figure 3E, R2=0.3735, *P*<0.01). Similarly, the RNA decay rates of Runx2 mRNA after transcription inhibition was dramatically increased but not affected by mutated FTO overexpression. Taken together, these findings suggested that FTO represses the expression of Runx2 through its m6A demethylase activity.

### Inhibition of FTO promotes bone formation of OVX mice *in vivo*

**Figure 4:**
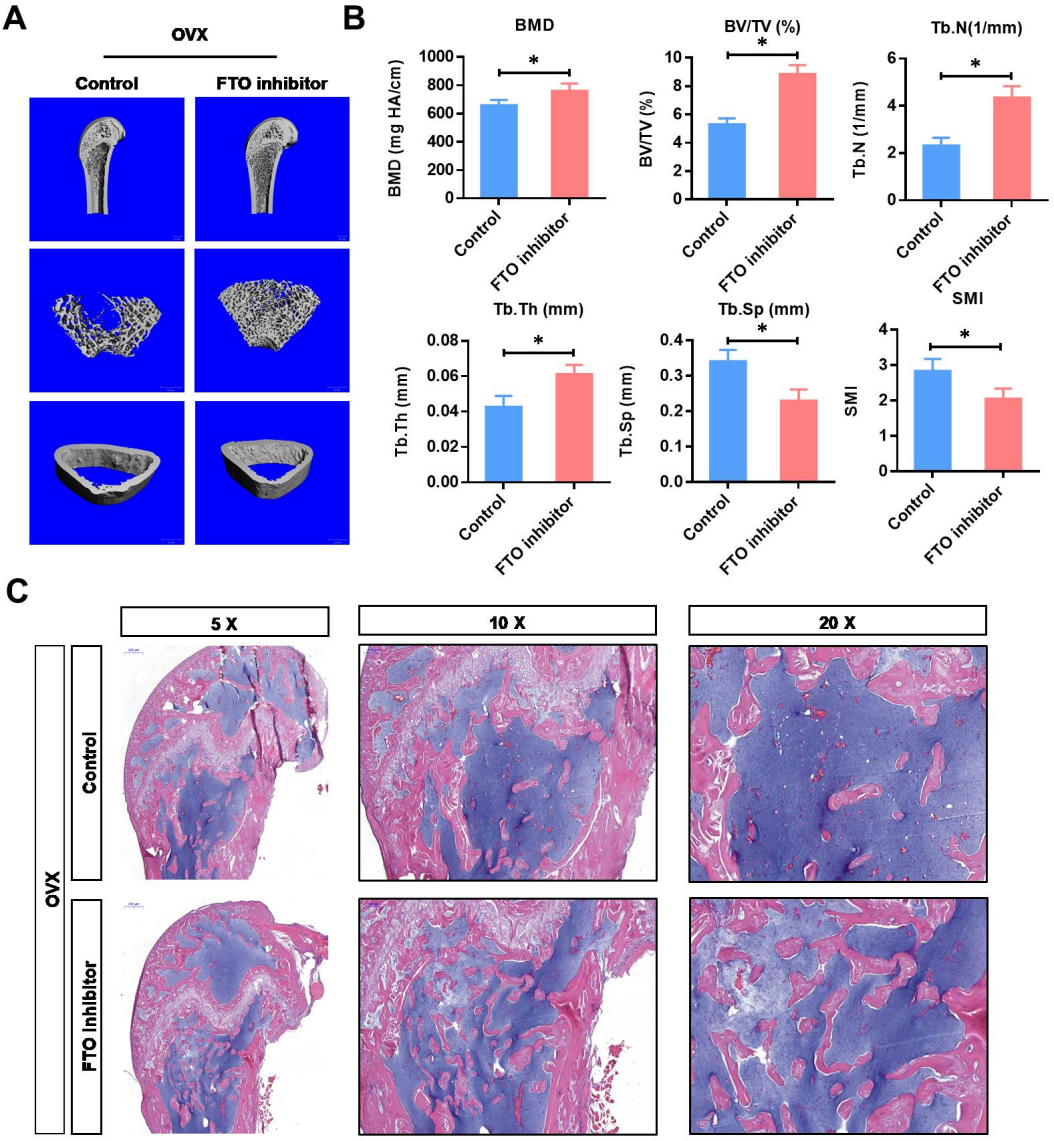
**Inhibition of FTO promotes bone formation of OVX mice *in vivo*.** (**A**) Representative images of μCT reconstructive images of tibial plateau in OVX groups with or without FTO inhibitor. (**B**) 3D structural parameters-BMD, BV/TV, Tb.N, Tb.Sp, Tb.Th and SMI-of tibial plateau by μCT in in OVX groups with or without FTO inhibitor. *P<0.05. (**C**) Representative hematoxylin-eosin staining images of tibial plateau showing bone volume in each group.

To investigate the critical function of FTO *in vivo*, we used the OVX animal model. As shown in Figure 4A, the BMD and BV/TV detected by μCT analysis were significantly increased in FTO inhibited OVX mice. In addition, FTO inhibition also increased the Tb.N and Tb.Th, while decreasing the Tb.Sp and SMI. Furthermore, the histological evaluation suggested that the overall area of stained bone from the FTO inhibited OVX group was significantly up-regulated compared with those in the normal OVX group, which suggests that the impaired development of new bone was reversed by FTO inhibition. Collectively, these results indicate that therapeutic inhibition of FTO may able to counteract the bone loss in osteoporosis.

## DISCUSSION

The m^6^A modification in Eukaryotic RNA has been reported to have a wide range of functional effects on homeostasis, and any disturbance of m6A levels may lead to dysfunction or disease [11, 13, 14, 18]. Disturbance of m6A levels disrupts the strictly controlled embryonic stem cell differentiation and impairs the functional homeostasis of spermatogenesis, T-cell development, and neurogenesis [19]. But the role of m^6^A in the develop of OP remains unclear. A recent study has consecutively revealed that knockout of METTL3 in BMSCs of mice disrupts cell fate and results in osteoporosis pathological phenotypes, uncovering an efficient and specific regulation of METTL3 mediated m6A on MSCs [20]. As the first identified RNA demethylase, FTO was closely related with increased body mass and obesity [11], and reported to regulate dopaminergic signaling in adipogenesis and leukemogenesis [12, 14].

In this study, these results provided the basis for understanding of the biological role of m^6^A demethylase FTO in OP development for the first time. Along with a significantly elevated level of m^6^A, the expression level of FTO was significantly increased in OP-BMSCs and OVX-BMSCs compared with normal BMSCs. *In vitro* study showed that FTO over-expression impaired the osteogenic differentiation ability of BMSCs. Furthermore, FTO inhibition prevents OVX induced osteoporosis, establishing the indispensability of FTO during the osteogenic differentiation in BMSCs and thereby ensuring skeletal health.

Runx2 is a critical transcription factor in osteoblast differentiation through inducing Sp7 [21]. Here, we found that FTO over-expression in BMSCs significantly decreased the methylated level of Runx2 mRNA and promoted the RNA decay rates, therefore decreased the expression level of Runx2. Importantly, over-expression of FTO with demethylase activity mutated (R96Q) failed to affect both the methylated level and RNA decay rates of Runx2. We predicted the potential m6A sites via SRAMP database (http://www.cuilab.cn/sramp/) and found 9 highly conserved m6A sites. Here, we demonstrated that FTO regulates the expression level of Runx2 mRNA via its m^6^A demethylase activity, which may be a valuable regulatory mechanism in osteoblast differentiation. However, as an extensive m^6^A demethylase, FTO may regulate the osteogenic differentiation of BMSCs from multiple angles and dimensions, which is needed to be revealed with further study.

In conclusion, our results demonstrate that RNA N6-methyladenosine demethylase FTO promotes osteoporosis through demethylating Runx2 mRNA and inhibiting osteogenic differentiation. These findings indicate that FTO mediated demethylation of Runx2 may be a potential target for treatment of OP.
